# Risk Assessment of Endometrial Hyperplasia or Endometrial Cancer with Simplified Ultrasound-Based Scoring Systems

**DOI:** 10.3390/diagnostics11030442

**Published:** 2021-03-04

**Authors:** Norbert Stachowicz, Agata Smoleń, Michał Ciebiera, Tomasz Łoziński, Paweł Poziemski, Dariusz Borowski, Artur Czekierdowski

**Affiliations:** 1 Chair and Department of Epidemiology and Clinical Research Methodology, Medical University of Lublin, 20-080 Lublin, Poland; agatasmolen@umlub.pl; 2 Center of Postgraduate Medical Education, Second Department of Obstetrics and Gynecology, 01-809 Warsaw, Poland; michal.ciebiera@gmail.com; 3 Department of Obstetrics and Gynaecology, Pro-Familia Hospital, 35-001 Rzeszów, Poland; tomasz.lozinski@pro-familia.pl; 4 Department of Obstetrics and Gynecology, Mińsk Mazowiecki County Hospital, 05-300 Mińsk Mazowiecki, Poland; poziemskip@gmail.com; 5 Clinic of Fetal-Maternal Medicine, Gynecology and Neonatology, Collegium Medicum, Nicolaus Copernicus University in Bydgoszcz, 85-067 Bydgoszcz, Poland; dariuszborowski@cm.umk.pl; 6 Department of Gynecological Oncology and Gynecology, Medical University of Lublin, 20-081 Lublin, Poland; arturczekierdowski@umlub.pl

**Keywords:** endometrium, cancer, hyperplasia, sonography, risk scoring

## Abstract

Background: Abnormal uterine bleeding (AUB) represents a common diagnostic challenge, as it might be related to both benign and malignant conditions. Endometrial cancer may not be detected with blind uterine cavity sampling by dilatation and curettage or suction devices. Several scoring systems using different ultrasound image characteristics were recently proposed to estimate the risk of endometrial cancer (EC) in women with AUB. Aim: The aim of the present study was to externally validate the predictive value of the recently proposed scoring systems including the Risk of Endometrial Cancer scoring model (REC) for EC risk stratification. Material and methods: It was a retrospective cohort study of women with postmenopausal bleeding. From June 2012 to June 2020 we studied a group of 394 women who underwent standard transvaginal ultrasound examination followed by power Doppler intrauterine vascularity assessment. Selected ultrasound features of endometrial lesions were assessed in each patient. Results: The median age was 60.3 years (range ± 10.7). The median body mass index (BMI) was 30.4 (range ± 6.0). Histological examination revealed 158 cases of endometrial hyperplasia (EH) and 236 cases of EC. Of the studied ultrasound endometrial features, the highest areas under the curve (AUCs) were found for endometrial thickness (ET) (AUC = 0.76; 95% CI: 0.71–0.81) and for interrupted endomyometrial junction (AUC = 0.70, 95% CI: 0.65–0.75). Selected scoring systems presented moderate to good predictive performance in differentiating EC and EH. The highest AUC was found for REC model (AUC = 0.75, 95% CI: 0.70–0.79) and for the basic model that included ET, Doppler score and interrupted endometrial junction (AUC = 0.77, 95% CI: 0.73–0.82). REC model was more accurate than other scoring systems and selected single features for differentiating benign hyperplasia from EC at early stages, regardless of menopausal status. Conclusions: New scoring systems, including the REC model may be used in women with AUB for more efficient differentiation between benign and malignant conditions.

## 1. Introduction

Abnormal uterine bleeding (AUB) and the differentiation of underlying endometrial lesions represent common diagnostic challenges in everyday gynecological practice. Most of the reasons for AUB are related to benign conditions such as changes in steroid hormone levels, uterine fibroids and various forms of endometrial hyperplasia (EH) [1,2]. Once the symptom is present, the clinicians’ priority is to detect whether or not it could be related to intrauterine malignant growth and next, to assess the options for the optimal management [3,4,5]. In addition, in postmenopausal women the risk of endometrial cancer (EC) is substantially increased and any uterine bleeding may be the first sign of this malignancy [6]. Advances in transvaginal ultrasound technology have significantly improved the ability of clinicians to discriminate between benign and malignant changes [7]. It is tempting to hypothesize that early EC detection may substantially improve survival rates in affected women [8].

The main advantage of ultrasound imaging is that in the hands of experienced examiners the method has the highest accuracy for the preoperative classification of intra and extrauterine lesions, both benign and malignant [9,10]. If increased endometrial thickness (ET) is found in postmenopausal women with uterine bleeding, the risk of EC substantially increases [11,12,13,14]. Most gynecologists recommend the use of one of the invasive procedures in postmenopausal women with ET larger than 4–5 mm at ultrasound examination [12,13,15]. However, in women with type II EC, ET below 3–4 mm might also occasionally be found [15]. Because of these limitations, ET should not be the only factor to be considered for cancer risk estimation in women with AUB [16].

Image resolution is also extremely important in the studies of endometrial lesion vascularity as transvaginal probes of most currently used ultrasound high-end scanners are sensitive enough to detect Doppler signal and blood flow even in very small tumor vessels [17,18]. It might be important, e.g., in cases of distinguishing between adenomyosis and EC [19]. In 2003, Alcazar et al. demonstrated that transvaginal ultrasonography with the use of power Doppler blood flow mapping was useful in differentiating benign from malignant endometrial lesions in women presenting with postmenopausal bleeding and abnormally thickened endometrium [7]. These authors also showed that power Doppler blood flow mapping was effective in diagnosing almost all cases of EC (97%), polyps in about 92% of cases, endometrial hyperplasia in 79% and endometrial cystic atrophy in 85% [7]. Moreover, as many as 81.3% of EC cases had multiple vessels detected within the endometrium and at the endometrial-myometrial interface. The results clearly indicated that important neoangiogenic processes occurred in EC within tumor tissue and the surrounding area and that tumor vascularity could be considered as a characteristic feature for this type of cancer.

In our early studies, we assessed the value of three-dimensional sonography in women with EH and EC and found that the assessment of endometrial volume along with tumor blood flow vascular indices could improve the diagnostic precision of the sonographic estimation of endometrial lesions in postmenopausal women [20,21]. Those findings were later confirmed by Odeh et al. in 2007 [22] and Galvan et al. in 2010 [23] who found that three-dimensional power Doppler analysis of tumor vascularization in EC was reproducible and correlated with some prognostic histological characteristics [22,23]. In their study from 2011, Epstein et al. used both two-dimensional and three-dimensional sonography in a group of 144 women with EC and found that the tumor characteristics were significantly associated with tumor stage, grade and size. In this study, advanced ECs more often presented tumors with mixed/hypoechoic echogenicity, a higher color score and multiple globally entering vessels. Conversely, early ECs were more often hyperechoic and had no color or a low color score at power Doppler examination. Despite encouraging results, three-dimensional sonography is still not widely used because it requires extensive training and expertise [18]. For instance, Green et al. found that the off-line assessment of myometrial or cervical invasion in women with EC using three-dimensional sonography had lower interrater reliability and lower accuracy than two-dimensional video clip assessment [24].

In order to facilitate the comparisons of studies conducted by various researchers and to develop new standards with a uniform clinical reporting system, the first international consensus statement on standardized terminology and ultrasound endometrial lesions classification was presented in 2010 by the International Endometrial Tumor Analysis (IETA) group [25]. The IETA group suggested standardized terminology for describing grayscale and color Doppler ultrasound images of the endometrium [26,27]. The classification system developed by the IETA includes a wide variety of variables, including echogenicity, endometrial midline, endometrial-myometrial junction, bright edge, presence of synechiae, intracavitary fluid and color Doppler analysis with vascular patterns. Additionally, the expert consensus proposed the use of contrast sonohysterography for the assessment of difficult focal endometrial lesions, and, especially, the outline of endometrial borders [26,27]. Although extremely precise and comprehensive, the use of IETA terminology cannot replace sonographer’s training and experience and it cannot compensate for poor ultrasound system quality. Moreover, if only the descriptive IETA features are used alone, they do not allow one to calculate the risk of EC and to estimate the confidence level of uterine lesion classification.

In recent years, the change in clinical workup towards a fast-track identification of EC in postmenopausal women based on the ultrasound transvaginal structured evaluation of the endometrial lesions has been observed. A clear advantage of the ultrasound-based predictive models in discriminating uterine cavity lesions in comparison with other methods, including subjective assessment, was demonstrated [28]. Observer-dependent scoring system seemed to perform well in the prediction of EC, e.g., with the area under the curve (AUC) of 0.95 (95% CI, 0.92–0.99) or 0.97 with the addition of gel infusion sonography (GIS) [29]. One such predictive system called the Risk of Endometrial Cancer scoring model (REC) was recently proposed [30,31]. The REC scoring system does not only evaluate ET, but also other important features, such as endometrial echogenicity, endomyometrial junction, type of lesion vasculature including the number and thickness of vessels, the so called “color splash” presence and, in a more advanced version, also the imaging of the endomyometrial junction with the use of GIS [30]. The prognostic accuracy of this model found in the initial studies was high, and it correctly identified malignancies in 9 out of 10 postmenopausal women with the ET of 5 mm or more [30]. In a more recent study from 2019, Dueholm et al. tested an even easier system that could be used for initial endometrial lesion differentiation [31]. The important component of this version is the assessment of the presence of any dominant vessels within the endometrial lesion. If found, the examiner is asked to answer whether the vessels are multiple, enlarged and whether the endomyometrial junction is interrupted. Another and more complex version of this scoring system also includes the use of gel infusion sonography (GIS). According to Dueholm et al., such a predictive model was characterized by the best performance in women with ET equal to 8 mm or more [31]. Compared to REC, the new system reduced the number of women who needed to be scored, while still correctly identifying most women with EC or atypical hyperplasia (AH) [31]. However, despite known advantages related to the use of saline infusion sonography (SIS) or GIS in the preoperative workup of women with AUB, a substantial risk of the seeding of malignant cells from the uterine cavity into the abdominal cavity during hysteroscopy as well as during contrast infusion sonography was proven [32,33].

The aim of the present study was to externally validate the predictive value of the recently proposed scoring systems including the REC for EC risk stratification as proposed by Dueholm et al. [29,31].

## 2. Materials and Methods

This was a retrospective cohort study of women with postmenopausal bleeding. Because of its retrospective design, patient’s agreement for the study participation was not necessary. The study was performed in three clinical locations: Department of Gynecological Oncology, Medical University of Lublin, Poland; Department of Obstetrics and Gynecology, Mińsk Mazowiecki County Hospital, Mińsk Mazowiecki, Poland; and County Health Center in Opole Lubelskie—Hospital in Poniatowa, Poland. Ultrasound examinations were carried out by three experts, OB/GYN consultants with high experience in gynecological ultrasound scanning. General Electric Healthcare E8 Voluson, Austria and Medison Accuvix V10, South Korea ultrasound devices equipped with 5–9 MHz vaginal probes were used in the study. All patients underwent a standard transvaginal ultrasound examination followed by power Doppler endometrial vascularity assessment. Patients with cervical cancer or uterine metastases, patients who had previous hysteroscopic endometrial resection and women with comorbidities and with no histopathological diagnosis were excluded from this study. Grayscale transvaginal ultrasound was performed with the visualization of the longitudinal and transverse sections of the uterus. The power Doppler examination was performed using the method predefined, standardized settings. Clinical and imaging features were entered into the study database and retrospectively assessed by one of the investigators (NS).

Our study was performed in accordance with good clinical practice and the Helsinki Declaration. All patients were asked and gave their consent to participate in the examinations. All personal data of patients are protected in accordance with the Regulation of the European Parliament and the Council of the European Union.

Selected ultrasound features of endometrial lesions defined by the IETA group were assessed in each patient. These features included: ET measured for both layers; internal endometrial echo structure (hyper-/hypo-/isoechoic; homo-/heterogeneity; cystic (yes/no); if cystic: regular/irregular cystic areas); subendometrial halo or switching zone (visualization (yes/no) and determination of the texture gap (yes/no)); endomyometrial junction (ordinary/irregular, homogeneous/inhomogeneous echo intensity, clear line (yes/no), broken (yes/no)) [25,34]. Color Doppler assessment of endometrial vascularity included the following features: vessels (presence (yes/no), presence of a dominant vessel (yes/no), if a dominant vessel: single or double (yes/no) (the term “presence of vascularity but not a single or double dominant vessel” was defined as: the presence of vascularity (yes) and dominant single vessel (no) and a dominant double vessel (no))); origin (focal/multifocal); many vessels (yes/no); branching (yes/no), if branching, orderly/disorderly; circular flow (yes/no) [31]. Moreover, as described earlier, we subjectively assessed other selected endometrial vascular features and these included: a high diameter of the vessels (yes/no), color splash (yes/no), densely packed blood vessels (yes/no).

**Figure 1:**
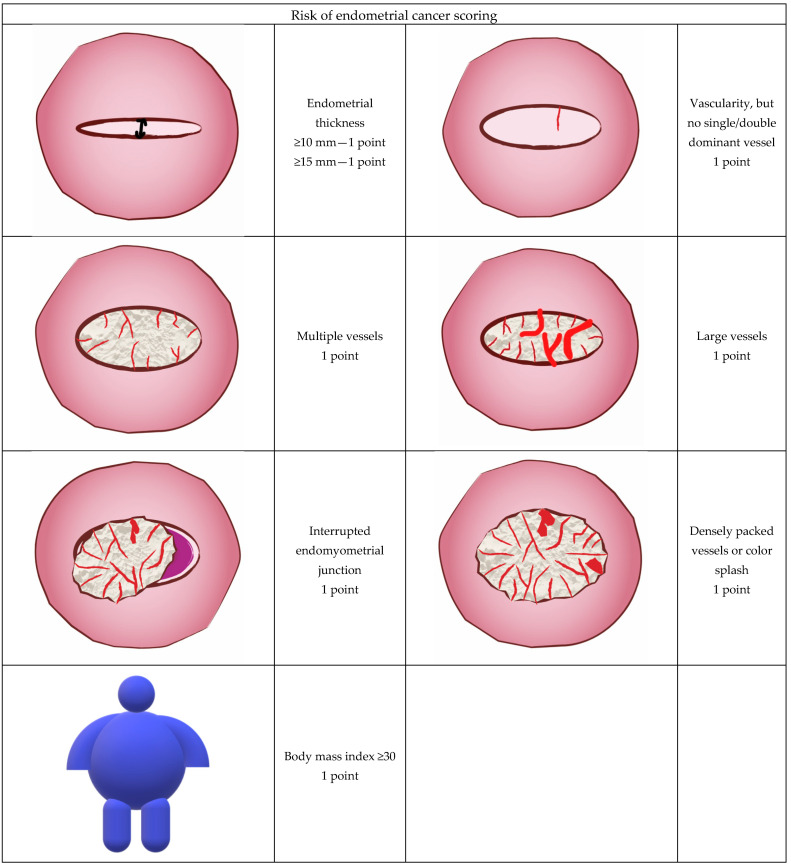
Ultrasound and color Doppler features used to calculate the Risk of Endometrial Cancer (REC) score in the current study.

Since intrauterine contrast sonography and, in particular, GIS were not performed during the studied period in participating centers we have chosen to compare predictive values of four non-GIS prognostic models proposed by Dueholm et al. [30,31]. According to their study protocol, GIS was performed when the endometrium was not clearly defined, the REC score obtained with transvaginal scan was indefinite or suspicion of a high probability of malignancy was undetermined by transvaginal scan [30]. Moreover, in a substantial proportion of women with postmenopausal bleeding there is some fluid in the uterine cavity and this amount may be sufficient to better evaluate the intrauterine cavity shape along with endometrial surface. Score A was calculated with the use of the following formula and parameters: interrupted endomyometrial junction (2 points if present) + vessels not stated as a dominant single/double vessel (1 point) + large vessels (1 point) and multiple vessels. The Doppler score was calculated and obtained by simple addition of the following Doppler parameters: vessels, but no dominant single/double vessel (1 point), multiple vessels (1 point), large vessels (1 point) [31]. Basic model included three variables: ET, Doppler score and interrupted endometrial junction (IEJ) and the predictive value was calculated according to formula originally presented by Dueholm et al. as follows. Basic model: z = −4.50 + 0.115 × (ET in mm) + 0.98 × (Doppler score) + 3.25 × (IEJ). Another tested model called model A was calculated as follows: Model A: z = −2.143787 + 1.186298 × (Doppler score) + 3.754607 × (IEJ). The probability of malignancy (p) was calculated as follows: p = 1/1 + e^−z^, where e = 2.718 [29]. The REC scoring system included: BMI (if ≥30 = 1 point), ET (if ≥10 mm = 1 point), (if ≥15 mm = 1 point), the presence of vascularity, but no single/double dominant vessel (if present = 1 point), multiple vessels (if present = 1 point), large vessels (if present = 1 point) and splashed/densely packed vessels (if present = 1 point), interrupted endomyometrial junction (if present = 1 point). Simple addition of these values constituted the REC score [29]. The graphic illustration of the sonographic features that were scored is presented in Figure 1.

Hysteroscopy or hysterectomy was performed following ultrasound examination in all patients. Histopathological evaluation served as a gold standard for the final diagnosis. After ultrasound examination, all patients underwent curettage of the uterine cavity or hysteroscopy (patients with the suspicion of endometrial polyp visible on ultrasound—19 women) in order to obtain material for histopathological examination. In total, 295 hysterectomies were performed, of which 280 were preceded by 4 hysteroscopies and 276 dilatations and curettage; 15 surgeries were performed without first step procedure. Our group also included 99 patients in whom we did not perform the hysterectomy; in these, 84 had dilatation and curettage and 15 had hysteroscopy. Three study groups were compared: women with the final diagnosis of simple endometrial hyperplasia (SH); patients with AH and women with EC. The EC group was further divided into two subgroups, i.e., women with endometrioid (EEC) and with non-endometrioid (NEC) cancers.

## 3. Results

From June 2012 to July 2020 we studied a group of 394 patients with AUB who underwent a standard transvaginal ultrasound examination followed by power Doppler intrauterine vascularity assessment. There were 289 (73.3%) postmenopausal women in the studied group. The median age of the studied patients was 60.3 years (range ± 10.7) and their median body mass index (BMI) was 30.4 (range ± 6.0). Table 1 presents a summary of selected demographic characteristics in the studied group of women.

Histological examination revealed 158 cases of endometrial hyperplasia including 103 (26.1%) cases of SH and 55 cases (14%) of AH. In 236 women with EC, there were 216 cases of EEC (54.8%) and 20 (5.1%) cases of NEC. Table 2 presents details of ultrasound measurements of ET and histological types of the studied endometrial lesions.

Interrupted endomyometrial junction was found in 62% of women with EC and in 51% of women with AH, but only in 7% of women with SH. Heterogeneous endometrium was observed in 86% of women with EC and in 94% of women with SH and AH. Scattered color Doppler signals in the endometrium, not defined as a single vessel, were found in 56% of women with EC, in 53% of women with AH and in 47% of women with SH. Large single vessels were observed in 5% of women with EC and AH, while in women with SH this feature was found in only 2% of cases. Densely packed vessels or “color splash” at power Doppler examination were observed in 9% of women with EC and in 2% of women with EH. Detailed characteristics of the selected ultrasound imaging features are shown in Supplementary Material.

**Figure 2:**
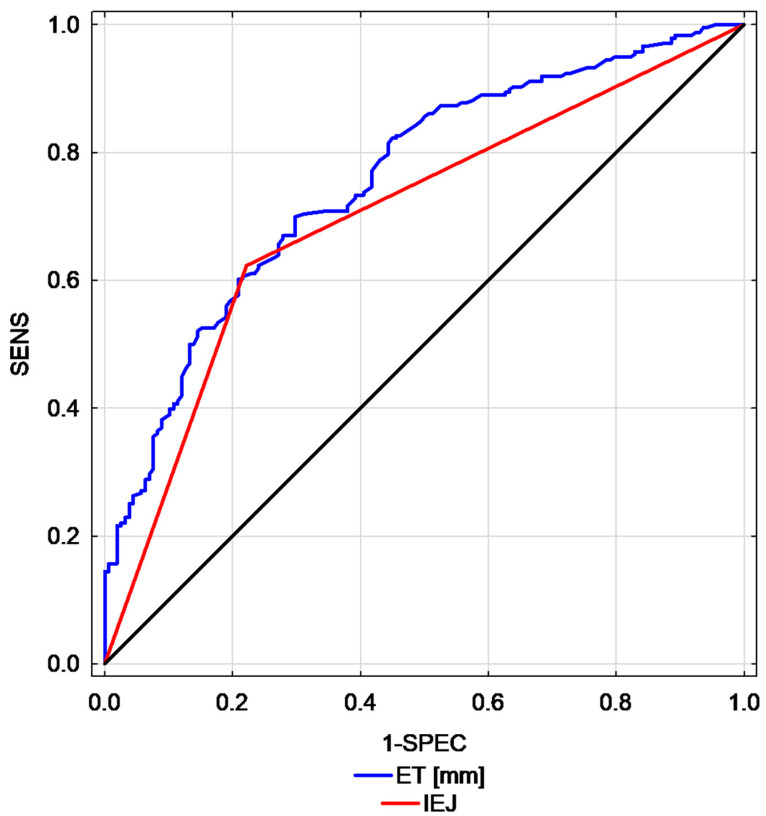
Comparison of receiver operating characteristic (ROC) curves calculated for endometrial thickness versus interrupted endomyometrial junction (endometrial thickness—ET, interrupted endomyometrial junction—IEJ, endometrial thickness—ET, interrupted endometrial junction—IEJ, sensitivity—SENS, specificity—SPEC).

Table 3 presents the comparison of EC/AH cases, demographic parameters and endometrial ultrasound features in the studied group. Of the studied ultrasound endometrial features, the highest AUCs were found for ET (AUC = 0.76; 95% CI: 0.71–0.81) and for interrupted endomyometrial junction (AUC = 0.70, 95% CI: 0.65–0.75). A graphic representation of the best parameters is presented in Figure 2.

**Figure 3:**
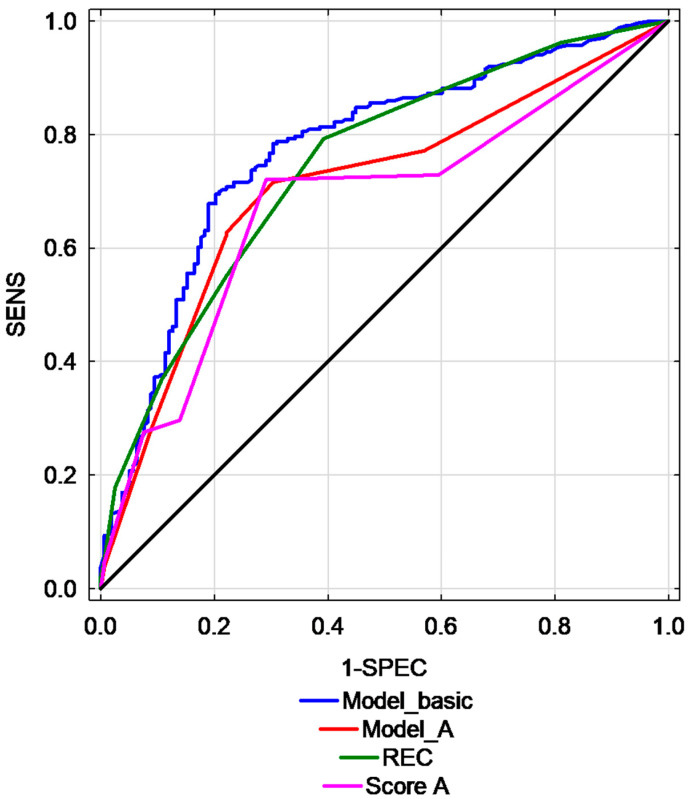
Comparison of ROC curves for different studied predictive models. (Risk of Endometrial Cancer scoring model—REC, sensitivity—SENS, specificity—SPEC, Model basic, Model A, Score A–various models).

Selected scoring systems presented moderate to good predictive performance in differentiating EC and EH. The highest AUC was found for REC model (AUC = 0.75, 95% CI: 0.70–0.79) and for basic model that included ET, Doppler score and interrupted endometrial junction (AUC = 0.77, 95% CI: 0.73–0.82). Table 4 presents the prognostic value of multivariate models used for the prediction of EC and AH. A graphic representation of this parameters is presented in Figure 3.

## 4. Discussion

Although only about 10% of women with AUB will have malignant tumors [31], the accurate imaging of endometrial lesions may be helpful in excluding or implementing further invasive diagnostic procedures to obtain material for histological evaluation of the endometrium [35,36]. EC when detected early may be effectively treated in the vast majority of cases [37,38]. The major limitation of transvaginal sonography in discriminating endometrial lesions is related to the fact that it is highly examiner-dependent and even with the same sonographer it may have a large inter-observer variability [39]. ET assessment with various cut-off levels was proposed and typically the thickness of both layers below 4–4.5 mm was regarded as the safe cut-off, practically excluding EC in the vast majority of cases [16,36,40].

In one of the first studies in 1990, Osmers et al. demonstrated that women with postmenopausal bleeding and ET of 4 mm or more should undergo a histological examination [41]. Similar results were then presented by Goldstein et al. (1991). Women with postmenopausal bleeding and an ET of 4 to 5 mm or less could be reliably excluded from having EC [40]. In a study by Karlsson et al. there were no cases of EC when the ET measured on ultrasound examination was thinner than 5 mm [42]. In 2003, Gull et al. demonstrated no cases of EC when the ET cut-off value of 4 mm was used, even during 10 years of clinical follow up. These authors also found that no increased risk of EC or AH was observed in women who did not have recurrent uterine bleeding [11].

Goldstein et al. have recently suggested that the use of ET measurements is often inappropriate and some clinicians might assume that all cases of thickened endometrium are abnormal and require investigation [43]. These authors stated that postmenopausal bleeding diagnosis should start with an ultrasound scan and if the endometrium is sufficiently distinct and thin, no further workup is obligatory [43]. It seems to be essential to include history-related criteria into differential diagnosis. In 2020, Clarke et al. found that in an initial bleeding episode in women for whom transvaginal ultrasound was recommended, ET (with the cut-off value of 4 mm) did not provide the optimal risk stratification in terms of the occurrence of cancer or atypical cells [44,45]. In such a case, the authors found out that patient’s age was another variable influencing the occurrence of endometrial lesions. Interestingly, patients younger than 60 with the ET equal to or lower than 4 mm were not diagnosed with cancer, while in women younger than 60 with the ET over 4 mm such lesions were reported in 8.4% of patients [45]. The authors suggested that future research should include various clinical and epidemiological data in the models for the best possible risk stratification in patients undergoing diagnostic workup to confirm or exclude EC [45]. Such conclusions were not new as similar ones were presented in 2011 by Hanegem et al., who also suggested that specific clinical data of a patient and epidemiological history might influence the selection of a suitable diagnostic method (e.g., ultrasound, hysteroscopy) [35]. Dueholm et al. did not include age in risk stratification factors, but they included BMI over 30 [30,31]. Similar to other studies, we found that BMI > 30 kg/m^2^ in our group of women with postmenopausal bleeding markedly increased the risk of developing EC.

It was suggested that the appearance of EC on ultrasound examination was significantly associated with tumor staging and tumor grading [18]. More advanced tumors in this study presented more often with hypoechoic or mixed echogenicity; they were also characterized by higher color score and the presence of multiple and larger intratumoral vessels. In contrast, less clinically advanced ECs were found to be hyperechoic either with no detected vessels or with scan vascularity only [18]. In 2014, Dueholm et al. presented a new simplified scoring system for the risk assessment [29]. Their first prognostic model included several ultrasound features like ET and echogenicity, presence of the middle echo, presence of cysts inside the endometrium, endometrial-myometrial border characteristics, presence of blood vessels, their number and type of branching, presence of intracavity lesions. Subsequent studies of this group revealed that their scoring system significantly increased the effectiveness of diagnosis of EC with an AUC = 0.95 [30,31].

Our results suggest that the predictive models that did not include GIS performed less well than in the original study [31]. We decided to study these models because GIS use is not always possible, especially in postmenopausal women who are diagnosed with uterine cervix adhesions. Our study indicated that regardless of the presence or absence of uterine bleeding in postmenopausal women, the thick endometrium on ultrasound examination can be more frequently found than previously assumed. As Goldstein et al. recently suggested, more complex evaluation is not necessary in such cases unless significant additional risk factors, like obesity, chronic hypertension or diabetes are present [43].

Despite many studies that have examined the role of ultrasound in endometrial lesions assessment during past years, the strength of the available evidence is still relatively low. The REC model was constructed using earlier sonography-based scoring systems with the addition of BMI, grayscale and Doppler ultrasound features and GIS findings. An original study has achieved significantly higher prognostic efficiency in comparison with the ET measurements alone or with the prior models. Replication of these unique studies with larger sample sizes might strengthen the evidence. The REC scoring system appears to be useful in EC risk stratification in women with postmenopausal bleeding. The use of this predictive model may contribute to limiting the number of unnecessary invasive intrauterine procedures. It needs to be mentioned here that, in a country like Poland, a considerable number of patients with AUB undergo curettage, despite the lack of abnormal ultrasonographic evidence or, in some cases, even without a prior ultrasound examination. In some situations it is due to the fact that currently some physicians are still not trained to use ultrasound devices or they use old ones which have been used for a very long time. Spotting and abnormal endometrium examined sonographically are indications for the procedure in patients treated with tamoxifen. According to new multicenter research conducted in countries like Italy, tamoxifen seems not to increase the risk of EC [46]. Obviously, the discussed issues were not observed in many centers, but for some patients unnecessary risk might be created [47]. Additionally, the performance of unnecessary procedures makes their availability lower for those women who really need them. Recommendations concerning the optimal management of such cases are still unavailable in Poland. We hope that such recommendations will be developed in the coming years. The system of the certification of gynecologists by medical societies results in higher levels of training in ultrasonographic techniques. It, in turn, would facilitate faster access to invasive methods for women who are at a higher risk and improve the flow of funds for healthcare in the area of gynecology and gynecological oncology.

Notably, numerous centers switched to the endoscopic diagnostics of AUB. This is justified, as hysteroscopic endometrial biopsy/resection is performed under direct visualization and is the only technique that allows for the selective biopsy of the targeted areas of the uterine cavity [48]. In the case of low-quality ultrasound or little experience in the assessment of endometrial lesions, hysteroscopy may be the best solution. Moreover, advanced equipment may in some cases facilitate the evaluation of the uterine cavity even in an outpatient setting [49]. However, it needs to be remembered that some infiltrating cancers of the endometrium do not present as a focal thickening of the endometrium, but present the direct infiltration into the myometrium, which might be overlooked through diagnostic hysteroscopy [19].

We believe that the strength of the presented study is the relatively large group of women with AUB and histologically proven endometrial hyperplasia or cancer. Conversely, such a group is unsuitable to make strong conclusions, which constitutes a limitation of the study. Therefore, we need to wait for the results of studies conducted in larger samples. This group was studied by experienced, certified sonographers with the use of adequate equipment. The main weakness of our study is related to its retrospective design. Another potential weakness is the fact that, as in the original publication of Dueholm et al. [29], we used a study population with a high prevalence of EC. This means that the used risk scoring systems might not be applicable in a low-risk population. However, the diagnostic performance in our patients with SH, AH and EC seemed to be comparable.

## 5. Conclusions

Mathematical risk scoring systems such as the REC or Score A are relatively new prognostic tools that can be used for identifying the presence of EH and EC, thus enabling initial risk stratification between benign and malignant intrauterine lesions. The REC model seems to be more specific in estimating the risk of EC in women with postmenopausal bleeding. In our retrospective study, the REC score was more accurate than ET measurements alone when used for differentiating benign hyperplasia from EC in early stages, regardless of patient’s menopausal status. Both REC and ET were favorable in differentiating EC and EH in the case of unknown pathology or in various types of endometrial lesions.

We propose that larger and collaborative projects be undertaken for a better and more reliable verification of the proposed models to aid in the better understanding of their role in EC prediction.

## Figures and Tables

**Table 1:** Selected demographic features of the studied group of women (body mass index—BMI).

Age (years)	60.3 ± 10.7
BMI (kg/m^2^)	30.4 ± 6.0
BMI (kg/m^2^) ≥ 30 kg/m^2^ (*n*)	180 [46%]
Menopausal status	
Postmenopausal (*n*)	289 [73.4%]
Premenopausal (*n*)	105 [26.6%]

**Table 2:** Selected features of studied women with the specific histological diagnosis (endometrial thickness—ET, endometrial cancer—EC, endometrial hyperplasia without atypia—SH, atypical endometrial hyperplasia—AH).

	*n*	%
All patients	394	100
Hyperplasia without atypia (SH)	103	26.1
Atypical hyperplasia (AH)	55	14
Endometrial Cancer (EC)	236	59.9
Endometrioid EC (EEC)	216	54.8
Non-endometrioid EC (NEC)	20	5.1
Stage 1	152	38.57
Stage 2	47	11.93
Stage 3	30	7.61
Stage 4	7	1.77
ET ≥ 8 mm	332	84.26
EC or AH in women with ET ≥ 8 mm (% of total number of EC/AH)	263	90.37

**Table 3:** Clinical and sonographic parameters used for the prognosis of endometrial cancer or endometrial hyperplasia in validation study (endometrial hyperplasia—EH, body mass index—BMI, transvaginal sonography—TVS, interrupted endomyometrial junction—IEJ, endometrial cancer—EC, atypical hyperplasia—AH, area under curve—AUC).

	*n*	AUC (95% CI)	Sensitivity (%)	Specificity (%)	Accuracy (%)	LR+	LR−
BMI	394	59 (53–69)					
BMI ≥ 30	180	58 (52–64)	52	65	57	1.47	0.75
Endometrial thickness	394	76 (71–81)					
Endometrial thickness ≥8	332	60 (54–66)	92	28	67	1.28	0.27
Endometrial thickness ≥10	299	65 (60–71)	88	42	70	1.53	0.28
Endometrial thickness ≥15	221	68 (63–74)	71	66	69	2.07	0.44
Heterogenic endometrium	350	54 (48–60)	14	94	46	2.28	0.91
Cystic endometrium	67	55 (49–60)	21	89	48	1.82	0.89
Interrupted endomyometrial junction (IEJ)	182	70 (65–75)	62	78	69	2.81	0.48
Vascularity, and more than one single or double dominant vessel (a)	208	53 (48–59)	56	51	54	1.14	0.87
Multiple endometrial vessels (b)	110	61 (56–67)	37	85	56	2.53	0.74
Large endometrial vessels (c)	17	51 (45–57)	5	97	42	1.61	0.98
Densely packed or color splash	25	54 (48–59)	9	98	45	4.91	0.92
Doppler score (a + b + c)	394	58 (53–64)					
Doppler score (a + b + c) ≥ 1	209	54 (48–59)	56	51	54	1.15	0.86
Doppler score (a + b + c) ≥ 2	115	60 (55–66)	37	83	56	2.18	0.76

**Table 4:** Scoring systems performance for the prediction of atypia or endometrial cancer (Area Under the Curve—AUC, LR+—positive likelihood ratio, LR−—negative likelihood ratio, endometrial thickness—ET, interrupted endomyometrial junction—IEJ).

*n* = 394	AUC (95% CI)	Sensitivity (%)	Specificity (%)	Accuracy (%)	LR+	LR−
Score A	68 (62–73)	72	71	72	2.47	0.40
REC	75 (70–79)	79	61	72	2.02	0.34
Basic Model [29] ET, Doppler score, IEJ	77 (73–82)	78	70	75	2.58	0.31
Model A [31] Doppler score, IEJ	71 (66–76)	72	70	71	2.36	0.41

Basic model: z = −4.50 + 0.115 × (endometrial thickness in mm) + 0.98 × (Doppler score) + 3.25 × (IEJ). Model A: z = −2.143787 + 1.186298 * (Doppler score) + 3.754607 × (IEJ) The probability of malignancy (p) was calculated as follows: p = 1/1 + e^−z^, where e = 2.718.

## Data Availability

The data used to support the findings of this study are available from the corresponding author upon request.

## Supplementary Materials

The following are available online at https://www.mdpi.com/2075-4418/11/3/442/s1. Table S1: Endometrial image parameters in relations to endometrial pathology.
