# Supplementary material for: Risk Assessment of Endometrial Hyperplasia or Endometrial Cancer with Simplified Ultrasound-Based Scoring Systems
Source: Diagnostics (Basel). 2021 Mar 4;11(3):442. doi: 10.3390/diagnostics11030442 (PMC8001089; doi:10.3390/diagnostics11030442)
Supplement: Supplementary file 1 [file diagnostics-11-00442-s001.pdf]

Supplementary Material Table. Endometrial image parameters in relations to endometrial pathology.

**Pathology**	**N**	**BMI**		**Endometrial thickness**		**Endo-myome-trial junction**	**Endometrial structure**		**Doppler findings in endometrium Vessels present AND**				**Doppler score**	
		**Mean [+/-SD]**<br>**Numbers >=30 kg/m^2 (%)**		**Mean [+/-SD]**<br>**Numbers >=15 mm (%)**<br>**Numbers >=10 mm (%)**<br>**Numbers >=8 mm (%)**		**Inter-rupted N (%)**	**Cystic N (%)**	**Hetero-genic N (%)**	**More than one single vessel N (%)**	**Multiple vessels N (%)**	**Large vessels N (%)**	**Densely packed or color splash N (%)**		**N (%)**
EEC	216	31.0	6.0	21.6	11.4	135 (63)	42 (19)	184 (85)	119 (55)	76 (35)	10 (5)	19 (9)	0	96 (44)
													1	43 (20)
													2	69 (32)
		111	51%	151	70%								3	8 (4)
				190	88%									
				200	93%									
NEC	20	30.5	5.6	21.7	10.8	12 (60)	7 (35)	18 (90)	12 (60)	11 (55)	2 (10)	3 (15)	0	8 (40)
													1	1 (5)
													2	9 (45)
		11	3%	16	80%								3	2 (10)
				18	90%									
				18	90%									
**Total EC**	**236**	**31.0**	**6.0**	**21.6**	**11.3**	**147 (62)**	**49 (21)**	**202 (86)**	**131 (56)**	**87 (37)**	**12 (5)**	**22 (9)**	**0**	**104 (44)**
													**1**	**44 (19)**
		**122**	**52%**	**167**	**71%**								**2**	**78 (33)**
				**208**	**88%**								**3**	**10 (4)**
				**218**	**92%**									

SH	103	29.1	6.0	11.4	[5.9]	7 (7)	5 (5)	99 (96)	48 (47)	11 (11)	2 (2)	1 (1)	0	55 (53)
													1	35 (34)
		32	9%	28	27%								2	13 (13)
													3	0 (0)
				52	50%									
				69	67%									
AH	55	30.1	6.2	15.3	7.2	28 (51)	13 (24)	49 (89)	29 (53)	12 (22)	3 (5)	2 (4)	0	26 (47)
													1	15 (27)
		26	47%	26	47%								2	13 (24)
				39	71%								3	1 (2)
				45	82%									
**Total EH**	**158**	**29.4**	**6.0**	**12.7**	**6.7**	**35 (22)**	**18 (11)**	**148 (94)**	**77 (49)**	**23 (15)**	**5 (3)**	**3 (2)**	**0**	**81 (51)**
													**1**	**50 (32)**
		**58**	**37%**	**54**	**34%**								**2**	**26 (16)**
				**91**	**58%**								**3**	**1 (1)**
				**114**	**72%**									
**Total**	**394**	**30.4**	**6.0**	**18.0**	**10.6**	**182 (46)**	**67 (17)**	**350 (89)**	**208 (53)**	**110 (28)**	**17 (4)**	**25 (6)**	**0**	**185 (47)**
													**1**	**94 (24)**
		**180**	**46%**	**221**	**56%**								**2**	**104 (26)**
				**299**	**76%**								**3**	**11 (3)**
				**332**	**84%**									
